# A Retrospective Performance Assessment of the Developmental Neurotoxicity Study in Support of OECD Test Guideline 426

**DOI:** 10.1289/ehp.11447

**Published:** 2008-08-12

**Authors:** Susan L. Makris, Kathleen Raffaele, Sandra Allen, Wayne J. Bowers, Ulla Hass, Enrico Alleva, Gemma Calamandrei, Larry Sheets, Patric Amcoff, Nathalie Delrue, Kevin M. Crofton

**Affiliations:** 1 Office of Research and Development, National Center for Environmental Assessment; 2 Office of Pesticide Programs, U.S. Environmental Protection Agency, Washington, DC, USA; 3 Syngenta CTL, Cheshire, UK; 4 Environmental Health Science Bureau, Health Canada, Ottawa, Canada; 5 Department of Toxicology and Risk Assessment, National Food Institute, Technical University of Denmark, Søborg, Denmark; 6 Dipartimento di Biologia Cellulare e Neuroscienze, Istituto Superiore di Sanità, Rome, Italy; 7 Bayer CropScience LP, Stilwell, Kansas, USA; 8 Environment, Health, and Safety Division, Organisation of Economic Co-operation and Development, Paris, France; 9 Office of Research and Development, National Health and Environmental Effects Research Laboratories, U.S. Environmental Protection Agency, Research Triangle Park, North Carolina, USA

**Keywords:** children’s health, developmental neurotoxicity, guideline, hazard assessment, Organisation of Economic Co-operation and Development, risk assessment, U.S. Environmental Protection Agency

## Abstract

**Objective:**

We conducted a review of the history and performance of developmental neurotoxicity (DNT) testing in support of the finalization and implementation of Organisation of Economic Co-operation and Development (OECD) DNT test guideline 426 (TG 426).

**Information sources and analysis:**

In this review we summarize extensive scientific efforts that form the foundation for this testing paradigm, including basic neurotoxicology research, interlaboratory collaborative studies, expert workshops, and validation studies, and we address the relevance, applicability, and use of the DNT study in risk assessment.

**Conclusions:**

The OECD DNT guideline represents the best available science for assessing the potential for DNT in human health risk assessment, and data generated with this protocol are relevant and reliable for the assessment of these end points. The test methods used have been subjected to an extensive history of international validation, peer review, and evaluation, which is contained in the public record. The reproducibility, reliability, and sensitivity of these methods have been demonstrated, using a wide variety of test substances, in accordance with OECD guidance on the validation and international acceptance of new or updated test methods for hazard characterization. Multiple independent, expert scientific peer reviews affirm these conclusions.

The purpose and intent of this retrospective performance assessment was to review the history of developmental neurotoxicity (DNT) testing. This review demonstrates the extensive scientific efforts, including basic neurotoxicology research, interlaboratory collaborative studies, expert workshops, and validation studies, that form the foundation for this testing paradigm. We also review the relevance, applicability, and use of the DNT study in human health risk assessment and the historical performance of the DNT study. This analysis was developed by an OECD expert group [Organisation for Economic Co-operation and Development (OECD) 2008a] in support of drafting the OECD DNT Test Guideline 426 (TG 426; OECD 2007) that satisfies current OECD validation criteria.

OECD validation criteria are described in Guidance Document 34 (GD34; OECD 2005a), which addresses the validation and regulatory acceptance of new or updated test methods for hazard characterization. They are based on the “Solna Principles” for validation and regulatory acceptance (OECD 1996b), but additionally emphasize the importance of flexibility and adaptability in the validation process without compromising scientific rigor.

GD34 (OECD 2005a) also provides concise definitions of related concepts such as accuracy, concordance, performance standards, predictivity, relevance, reliability, repeatability, reproducibility, sensitivity, and specificity. The terminology and definitions presented in GD34 (Annex 1) were used in the DNT review process; however, individual studies may have varied slightly in the definition of terms.

The first DNT guideline was developed by the U.S. Environmental Protection Agency (EPA) and has been subjected to numerous validation studies and rigorous peer reviews over the years. The U.S. EPA has deemed the method validated for its regulatory purposes. As described herein, extensive supportive materials for the relevance, reliability, and overall performance of the DNT study are available. Until recently, only the U.S. EPA DNT guideline has been available for testing laboratories. The new OECD TG 426 (OECD 2007) DNT guideline will fill a regulatory gap for OECD member countries. This review summarizes the considerable work that has been performed in the development of the DNT study and provides the rationale for the regulatory acceptance of TG 426 as a new OECD test guideline.

The U.S. EPA DNT guideline (U.S. EPA 1998), the prototype for TG 426, was founded upon an extensive scientific database. This includes interlaboratory validation studies, such as the Collaborative Behavioral Teratology Study (CBTS), which was conducted in the mid-1980s. A separate group of experts at the Williamsburg Workshop (Kimmel et al. 1990) agreed that the methods in the DNT study are sensitive to known human developmental neurotoxicants. An expert consultation meeting conducted in 2000 (OECD 2003) discussed issues on validation, especially of individual test components versus the whole DNT test method. In doing so, they reviewed the extensive history of international validation, peer review, and evaluation of DNT methods contained in the public record. Experts agreed that individual assays of the DNT test method have been shown to be relevant, reliable, and sensitive and that extensive information demonstrates the validity of individual components of the DNT test method (OECD 2003).

The field of developmental neurotoxicology evolved from the disciplines of neurotoxicology, experimental and development psychology, and developmental toxicology, through an extensive history of scientific research and regulatory consideration. Developmental toxicity is defined in GD43 (OECD 2008b), which states that effects may result from either prenatal or postnatal exposure, may manifest at any life stage, and may be expressed as functional deficits.

The DNT study is a specialized type of developmental toxicity study designed to screen for adverse effects of pre- and postnatal exposure on the development and function of the nervous system and to provide dose–response characterizations of those outcomes. The U.S. EPA and OECD DNT guidelines recommend administration of the test substance during gestation and lactation. Cohorts of offspring (typically rat) are randomly selected from control and treated litters for evaluations of gross neurologic and behavioral abnormalities during postnatal development and adulthood (OECD 2007; U.S. EPA 1998). These include assessments of physical development, behavioral ontogeny, motor activity, motor and sensory function, learning and memory, and postmortem evaluation of brain weights and neuropathology.

## History of DNT Test Guideline Development

The evolution of DNT studies has its roots in scientific publications that began to appear in the early 1960s; the science has continued to develop over the past four decades. An extensive scientific literature, composed of studies evaluating the potential for physical, pharmaceutical, and environmental agents to affect the development and function of the nervous system after prenatal and early post-natal exposure, provides a strong foundation for guideline development, implementation, and validation. Table 1 lists some of the key contributions to the development of the DNT guidelines. Table 2 briefly summarizes the history of U.S. EPA and OECD DNT guideline development. Although prenatal developmental toxicity test guidelines have existed for some time (e.g., OECD 1983), the first regulatory protocol designed to evaluate DNT was developed and implemented by the U.S. EPA in support of hazard evaluation for specific solvents (U.S. EPA 1986), and a DNT guideline applicable to the evaluation of both toxic substances and pesticides was finalized in 1991 (U.S. EPA 1991). In 1998, it was revised (U.S. EPA 1998) as part of a broader U.S. effort to harmonize testing guidelines within U.S. EPA program offices and with the OECD.

In 1995, the OECD initiated the development of the DNT TG 426 (OECD 1995). The first draft of TG 426 was prepared after an expert consultation meeting (OECD 1996a), using the U.S. EPA DNT guideline as the design template, and addressed a number of important issues and recommended improvements. The draft TG 426 was distributed for comment in 1998, and significant technical issues identified by this review (e.g., the optimal duration of treatment, direct dosing of preweaning rodents, and conduct of morphometric evaluations) were further discussed at an expert consultation meeting in 2000 (OECD 2003). A revised draft was subsequently circulated for review, and comments from OECD member countries were addressed at a 2005 expert consultation meeting (OECD 2005b). The final version of TG 426 was adopted by the OECD Council in 2007 (OECD 2007).

In the context of toxicologic screening and testing to support human health risk assessment and chemical regulatory activities, the DNT study fills an information requirement that is not satisfied by other OECD test guidelines. Notably, it is the only test guideline that includes functional, behavioral, and anatomical evaluations of the nervous system at multiple time points, in test subjects that were exposed to test substance during critical pre- and early postnatal periods of nervous system development. This test method has been used extensively in the past two decades on a wide variety of chemicals (Table 3).

## Scientific Basis of DNT Guideline

The test methods recommended in the DNT guideline have been extensively reviewed and evaluated over the last 25 years. This has included the conduct of a number of meetings and collaborative studies involving experts from academic, industry, regulatory, and public interest groups. Pivotal influences and key events in the history of the development of the DNT guideline (Table 2) include both research on test methods development and efforts to characterize and document the sensitivity, reliability, and performance of the test methods, including a number of intralaboratory collaborative efforts. In the 1970s, a series of studies were conducted in which rats were developmentally exposed to a variety of xenobiotics and subsequently tested during postnatal development using a battery of neurobehavioral tests (Butcher and Vorhees 1979; Vorhees et al. 1979). Other laboratories used behavioral and histologic batteries, focusing on sensory and motor function, in adult rodents exposed to a wide variety of neurotoxicants (Pryor et al. 1983; Tilson et al. 1979). A large body of research has provided an immense database on the ability of the functional observational battery to detect and characterize the effects of drugs and environmental chemicals in adult and developing animal models (Gad 1982; Irwin 1968; Moser et al. 1988). This early work was followed by wide-ranging efforts to characterize the specificity of these test methods and the impact of both organismal and experimental factors (e.g., noise, species, strain, gender, test history) (Gerber and O’Shaughnessy 1986; Levine and Butcher 1990; MacPhail et al. 1989; Spencer et al. 1993). Ultimately, the result of more than 30 years of work in this area is a consensus opinion of neuro toxicologists that proper use and interpretation of the data derived from these test methods provide unique insight into the impact of xenobiotics on the developing and adult nervous system [Cory-Slechta et al. 2001; International Programme on Chemical Safety (IPCS) 2001; Tyl et al. 2008].

The development of test methods in neurotoxicology also includes a long history of efforts to characterize the interlaboratory reliability and sensitivity of the test methods now included in the DNT study design. An article comparing a learning and retention method among three laboratories (Butcher et al. 1979) was followed by the CBTS (Buelke-Sam et al. 1985; Kimmel and Buelke-Sam 1985) and the “Williamsburg Workshop” on qualitative and quantitative comparability of human and animal DNT (Kimmel et al. 1990). These efforts addressed various aspects of DNT study design and conduct, providing a sound scientific basis for the test method and its use in hazard evaluation. Since the publication of the U.S. EPA DNT guideline (U.S. EPA 1991), a continued scientific effort has reviewed and updated methodologies, for neurotoxicology in general and for developmental neurotoxicology in particular. Examples of such reviews include the IPCS collaborative study on neurobehavioral screening methodologies (MacPhail et al. 1997), an International Life Sciences Institute (ILSI) Risk Science Institute (RSI) workshop on Developmental Neurotoxicity and Risk Assessment (Mileson and Ferenc 2001), a collaborative study on neurobehavioral screening in 11 Japanese laboratories (Okazaki et al. 2003), and a Behavioral Test Methods Workshop (Slikker et al. 2005). Descriptions of each of these efforts and their contributions to the scientific basis for DNT testing follow.

### The CBTS

Several of the test procedures developed in early behavioral teratology studies underwent validation in a large interlaboratory effort, the CBTS. This project characterized the performance of a standardized neurodevelopmental test battery in six different laboratories after *in utero* and lactational exposure to two known neurotoxicants, methylmercury and amphetamine. The study examined the intra- and inter-laboratory reliability and sensitivity of several behavioral test methods and the effects of a number of other litter- and gender-related variables. The peer-reviewed publications that resulted from the CBTS included descriptions of the background and overview (Kimmel and Buelke-Sam 1985), protocol and test procedures (Adams et al. 1985b), data entry and test systems (Adams et al. 1985c), preliminary research (Adams et al. 1985a), statistical approach (Nelson et al. 1985), results (Buelke-Sam et al. 1985), and implications, current applications, and future directions (Kimmel et al. 1985). Additionally, the results of a workshop held to review the CBTS data were published (Butcher and Nelson 1985; Geyer and Reiter 1985; Kutscher and Nelson 1985; Sobotka and Vorhees 1985; Tilson and Wright 1985). In a corollary study, many end points in the CBTS study were compared within one laboratory to an additional set of behavioral end points, named the Cincinnati Test protocol, using both methylmercury and -amphetamine (Vorhees et al. 1985a, 1985b). The conclusion was that the effects of methylmercury were detected by both the CBTS end points and some of the additional end points in the Cincinnati Test protocol (Vorhees 1985c). The CBTS showed that replicability of data among laboratories using a standardized protocol was excellent and that both positive effects (e.g., with methyl-mercury exposure) and the lack of effects (e.g., after low-level amphetamine exposure) were replicable. The CBTS also demonstrated that the DNT test procedures were sufficiently sensitive; no more than a 5–20% change from control values was required to detect an effect.

### The European Interlaboratory Collaborative Study

In the 1980s, the European Interlaboratory Study Group on Behavioural Teratology conducted a study of behavioral test methods (Alder et al. 1986; Elsner 1986; Elsner et al. 1986; Schreiner et al. 1986; Suter and Schon 1986). Three laboratories, one each from industry, academia, and government, tested animals perinatally exposed to methylmercury. The results indicated that behavioral tests were more sensitive than reproductive end points and that automated procedures and measures aimed at specific functional capacities were more sensitive than nonspecific behavioral measures (Elsner et al. 1986, 1988).

### The Williamsburg Workshop

In 1989, the U.S. EPA held a workshop on the Qualitative and Quantitative Comparability of Human and Animal Developmental Neurotoxicity (also known as the “Williamsburg Workshop”) to provide scientific input into DNT protocol design and to evaluate its appropriateness for use in risk assessment (Kimmel et al. 1990). Expert scientists from government, industry, public interest groups, and academia reviewed a range of representative chemicals and environmental exposures, including drugs (cannabis, cocaine, methadone, and phencyclidine) (Hutchings 1990), ethanol (Driscoll et al. 1990), the anticonvulsant phenytoin (Adams et al. 1990), and environmental contaminants such as methylmercury (Burbacher et al. 1990), lead (Davis et al. 1990), polychlorinated biphenyls (Tilson et al. 1990), and ionizing radiation (Schull et al. 1990). Based on data available for these known human developmental neurotoxicants, the workshop participants concluded that DNT methodologies were adequate for detecting DNT. A number of specific issues directly relevant to design and usefulness of DNT studies were extensively evaluated by participants (Buelke-Sam and Mactutus 1990; Levine and Butcher 1990; Stanton and Spear 1990; Tyl and Sette 1990). Additionally, workshop participants addressed the relationship between biologic end points specified by DNT guidelines and adverse findings observed in humans after exposure to the developmental neurotoxic agents under consideration. A major conclusion of the workshop was that the DNT protocol would have identified each of the agents presented at the workshop as a potential developmental neurotoxicant (Francis et al. 1990), although the critical effects and the dose at which the effects were observed could vary across species. The workshop participants also concluded that the laboratory animal is an adequate surrogate for humans because many of the biologic and behavioral mechanisms underlying these neurologic functions are shared between humans and laboratory animals. The predictive power of DNT guideline studies was attributed largely to the scope of neurobehavioral and neuropathologic tests used that can evaluate neurologic functions across multiple domains (i.e., sensory, motivational/arousal, cognitive, and motor).

### Collaborative studies of the Japanese Teratology Society

The Japanese Teratology Society established the Behavioral Teratology Meeting as a satellite meeting of the Japanese Teratology Society in 1982. This group sponsored a number of collaborative studies conducted primarily by pharmaceutical industry, and contract laboratories (Tanimura 1985). The first effort involved 21 institutions that investigated the effects of parametric variables (water temperature, number of trials) on performance in a water T-maze and two-way shuttle box (Mizutani 1984). This was followed by a larger study involving 46 laboratories that investigated the effects of six chemicals (chlorpromazine, ethanol, hydroxyurea, methylazoxymethanol, phenylalanine, and vitamin A) (Mizutani 1985). These studies demonstrated that the T-maze test was reliable, but possibly not as sensitive as needed, and suggested the need for more complicated learning paradigms for this method (Tanimura 1986). Workshops were then held between 1988 and 1990, with three subgroups: reflexes and sensory function, activity and emotionality, and learning (Tanimura 1992). Subsequently, a core battery test draft for behavioral developmental toxicity was proposed. Its utility was examined with three posi tive behavioral teratogens during 1993–1997: phenytoin (Fukunishi et al. 1998; Tachibana et al. 1996), retinoic acid (Nishmura et al. 2001), and nicotine (Tachibana et al. 1998). The numbers of participating laboratories were 32, 28, and 18, respectively. It was concluded that the proposed core battery of tests is useful as a method to detect postnatal developmental disorders, including behavioral dysfunction, in rats.

### The IPCS Study

The IPCS collaborative study was an interlaboratory evaluation of neurobehavioral screening methodologies used in adult and DNT studies (MacPhail et al. 1997; Moser et al. 1997e). A total of eight laboratories participated in proficiency studies (Moser et al. 1997a), and the full study evaluated seven neurotoxic positive control chemicals [triethyl tin, acrylamide, parathion, *p*,*p*′-dichlorodiphenyltrichloroethane (DDT), toluene, *N*,*N*′-methylene bisacrylamide, and lead acetate] in adult male rats (Moser et al. 1997b, 1997c). The study examined variability associated with the test methods and reasons for differences. The overall conclusion of this extensive study was general “agreement across laboratories in terms of their ability to detect dose-related changes in behavioral end points with prototypic neurotoxic agents” (Catalano et al. 1997). The study results were also reviewed at a workshop held in 1995 in Capri, Italy (Tilson et al. 1997) and were presented in a symposium at the 1996 meeting of the Society of Toxicology (Moser et al. 1997d).

### ILSI workshop on DNT testing

In 1999, ILSI established a working group of scientists from government, industry, academia, and nonprofit nongovernmental organizations (Mileson and Ferenc 2001) that was charged with evaluating revisions to the published U.S. EPA DNT guideline that were also included in the draft OECD TG 426. Some of these changes were implemented by the U.S. EPA Office of Pesticide Programs (OPP) when it issued Data Call-In notices for organophosphate pesticides with tolerances (U.S. EPA 1999b). The revisions included extension of the offspring dosing period through to the age of weaning, ensuring that the pups are exposed to the test substance, increasing the number of offspring evaluated neuropathologically, and collecting chemical-class–specific biomarker data. The extension of the dosing period during the lactation period raised several issues, specifically in the areas of pharmacokinetic/toxicokinetic data needs, behavioral testing, and neuropathologic evaluation. Overall, the working group agreed that the current DNT test protocol was based upon solid scientific principles and experience, that there were opportunities to revise and improve some aspects of the U.S. EPA guideline study, and that further research would be valuable in providing the scientific basis for development of TG 426 (Cory-Slechta et al. 2001; Dorman et al. 2001; Garman et al. 2001; OECD 2003). Further considerations of methodologic issues related to the conduct of the DNT study include an ILSI workshop on the direct dosing of preweaning mammals. This workshop culminated in a monograph on direct dosing that has broad application to study design for many areas of research, for example, pharmaceuticals, environmental pollutants, and academic research (Moser et al. 2005; Zoetis and Walls 2003).

### The Japanese Interlaboratory Study

An interlaboratory evaluation of neurobehavioral screening methodologies (used in DNT studies as well as adult neurotoxicity studies) was carried out by 11 safety research laboratories in Japan (Okazaki et al. 2003). The study examined technical problems in evaluating the neurotoxic potential of chemicals, conducting a variety of neurobehavioral tests on rats after either acute or repeated (28-day) exposure to acrylamide or 3,3′-iminodiproprionitrile. All laboratories detected neurotoxicity of both chemicals. The report identified interlaboratory differences in test method sensitivity and concluded that it is important to standardize the methods and criteria and to improve observers’ skills (Okazaki et al. 2003).

### The Behavioral Test Methods Workshop

In 2003, a workshop was conducted to discuss experimental procedures and practices that could help enhance the utility of behavioral data as a reliable index of neurotoxicity and in the safety evaluation of chemical substances (Slikker et al. 2005). Workshop participants included individuals from all sectors of the neuroscience community: academia, government, testing laboratories, industry, and nonprofit nongovernmental organizations. Overall conclusions from the workshop were that consensus can be reached on the fundamentals of behavioral assessment and that aspects such as experimental design, test method selection, training of technical staff, validation, control of confounding factors, data variability, data analysis, and data interpretation should be carefully considered in the planning and conduct of behavioral safety assessment (Slikker et al. 2005).

In summary, the scientific basis of the DNT test method has been subjected to an extensive history of international validation, peer review, and evaluation that is contained in the public record. Through the various collaborative efforts and workshops that have been conducted, a number of important conclusions have been drawn. The individual test methods used in the DNT study have been found to be highly relevant for characterizing health risks of neurotoxic chemicals and to be based on solid scientific principles and experience. Using exposures to known human developmental neurotoxicants, the DNT study has been shown to adequately identify the potential for adverse effects of chemical exposure on neurologic development. The intra- and interlaboratory reproducibility, reliability, and sensitivity of the DNT test method has been established, using a variety of test substances.

## Use of the DNT Study in Risk Assessment

There is a regulatory need for DNT testing to support risk assessments in OECD member countries. Many pesticides and other chemicals are known to affect the adult nervous system, and there are concerns regarding the potential for DNT after early-life exposures to these substances [National Research Council (NRC) 2000]. This is particularly important because unique behaviors and activities of children place them at greater risk for increased exposure to xenobiotics by multiple routes (Brent et al. 2004; Weiss et al. 2004). The call for a more rigorous assessment of the potential for DNT has been issued by scientists from multiple and diverse sectors with an interest in public health protection.

An examination of the historical and potential uses of the DNT study in risk assessment is critical to an overall evaluation of its value in protecting human health. Currently, the largest collection of DNT guideline studies resides with the U.S. EPA OPP, which has obtained information on DNT for specific pesticides to satisfy regulatory mandates (Food Quality Protection Act of 1996). The U.S. EPA has furthermore engaged in an ongoing scientific analysis and discourse regarding the conduct of DNT studies, the interpretation of the data from these studies, and their regulatory impact.

A review of 12 DNT studies (Makris et al. 1998) evaluated by the U.S. EPA Office of Prevention, Pesticides, and Toxic Substances (OPPTS) in support of the registration and/or use of nine pesticides and three solvents was presented to a Scientific Advisory Panel in 1998 (U.S. EPA 1999a). For the nine pesticides examined, the analyses demonstrated that the DNT study includes sensitive end points that are not examined in any other test guideline, including prenatal developmental, reproduction, and neurotoxicity studies (OECD 1983, 1997, 2001), thereby enhancing detection of neurobehavioral and neuropathologic effects in offspring after exposure during sensitive periods of neurologic development. As a result, the DNT study, when present in a chemical database, is often identified as a sensitive study and an important source of quantitative and qualitative information for risk assessment.

DNT data have been shown to be valuable in the selection of end points and doses for risk assessment (Makris et al. 1998; Rowland et al. 2007). As might be expected of a study that uses short-term exposures (~ 25–40 days) during development, where a single exposure during a critical period may result in developmental insult (Rice and Barone 2000; Rodier 1980, 1986, 1994), the predominant use of the DNT study in pesticide risk assessment has been for acute (single dose) reference doses (RfDs) and for short-term (1–30 days) and intermediate-term (1–6 months) nonoccupational exposures, which are especially applicable to risk assessments for children. Results from DNT studies have also been used in calculating a chronic RfD for lifetime exposure to a toxicant when it has been shown to be the most sensitive study in the toxicology database.

A survey of the use and value of neurobehavioral assessment in safety evaluation was also conducted by Middaugh et al. (2003). This survey included the results of multinational studies conducted since 1990 on 174 compounds, including pharmaceutical (81%), agricultural (7%), industrial (1%), or undefined (10%) substances. Notably, the neurobehavioral screening conducted for pharmaceuticals is generally composed of developmental and behavioral assessments of second-generation (F_1_) offspring but does not address all of the end points assessed in a guideline DNT study. Nevertheless, this review found that F_1_ behavioral parameters along with other parameters defined the chronic no observed effect level (NOEL) in 17 of 113 (15%) and solely defined the NOEL in 3 of 113 (2.6%) of the studies examined. The conclusion was that F_1_ behavioral parameters sometimes improved on the standard toxicologic measures of hazard identification, providing information on outcomes not addressed by other standard measures of toxicity.

By early 2006, a review of regulatory and published sources revealed that approximately 114 DNT studies had been completed using either the U.S. EPA guideline or the draft OECD guideline (Table 4). The list of agents in Table 4 demonstrates the extensive history and experience regarding the conduct and interpretation of DNT studies. The outcomes of these efforts do not comprise a focused attempt to validate the study protocol or specific end points. In fact, a few of these studies did not include all the end points recommended by U.S. EPA or OECD guidelines.

As of August 2006, approximately 75 DNT studies had been submitted to the U.S. EPA OPP in support of pesticide registration. A preliminary survey of the use of DNT studies in risk assessment in OPP was conducted in March 2007 (Rowland et al. 2007). For 58 of the 75 pesticide chemicals, a DNT study had been considered in the weight-of-evidence review of the toxicology database. The DNT study was used to select end points and doses for risk assessments for eight of those chemicals. Importantly, for four of the eight DNT studies, the critical effects either included or were solely based upon off-spring behavioral and neuropathologic parameters that are not evaluated in other guideline studies (i.e., motor activity, auditory startle habituation, learning and memory, and morphometric analysis). This is consistent with the conclusions of the earlier retrospective analysis (Makris et al. 1998) and provides further evidence of the sensitivity of the DNT study in identifying adverse effects in the young and the important role of DNT studies in human health risk assessments.

In addition to using DNT data for regulatory decisions, some regulatory agencies have also, on a case-by-case basis, incorporated an additional database uncertainty factor into their regulatory decisions because of the absence of DNT data. This approach reflects regulator views that DNT data are valuable in refining permissible exposure levels and that the absence of these data can increase the uncertainty about the toxicity of the chemicals (U.S. EPA 2002a, 2002b).

Recent reviews have examined specific end points across multiple guideline DNT studies, to demonstrate the value of current methods in hazard characterization and explore further opportunities for methodologic refinement. U.S. EPA scientists have conducted cross-laboratory comparisons of methodologies and results from DNT studies submitted to OPP (Crofton et al. 1991, 2004; Makris et al. 2005, 2006; Raffaele et al. 2003, 2004, 2005, 2006; Sette et al. 2004). Kaufmann and Groters (2006) reviewed the neuropathologic assessments in DNT studies, summarized practical aspects in planning neuropathologic assessments, and highlighted the value of morphology data in reference to both the concurrent behavioral assessments and use in assessing risk. A multisector ILSI RSI workshop examined the evaluation and interpretation of neurodevelopmental end points for human health risk assessment and addressed public health considerations, data interpretation, data variability, positive control data, and statistical analysis (Crofton et al. 2008; Fenner-Crisp et al. 2005; Holson et al. 2008; Raffaele et al. 2008; Tyl et al. 2008).

These various review efforts and resulting publications provide transparent decision criteria for the analysis and interpretation of DNT test results, in accordance with the principles described in GD34 (OECD 2005a). Additionally, they demonstrate test method reliability, reproducibility, and relevance, which is attributable in part to the high level of standardization of the test methods that are recommended in the test guideline.

## Future Activities

Although the overall performance of the DNT test method and its ability to detect effects of concern from a regulatory perspective have been well established, the recent increase in the number of regulatory DNT studies being conducted has refocused attention on this test method. Although some argue that specific tests are insensitive (e.g., assessment of cognitive and sensory dysfunction), others suggest that the tests are overly sensitive and have a high rate of false positives (U.S. EPA 1995; Claudio et al. 1999, 2000; Cory-Slechta et al. 2001; U.S. EPA 2006). Diverse groups have advocated increased testing for DNT (Andersen et al. 2000; Grandjean and Landrigan 2006; NRC 1992, 1993; Nelson 1986; Office of Technology Assessment 1990; Stein et al. 2002; Vorhees 1986). There have also been calls to include evaluations of end points not currently assessed, such as social behavior (Cory-Slechta et al. 2001), pharmacokinetics and neurochemistry (Andersen et al. 2000; Dorman et al. 2001), and changes during senescence (Cory-Slechta et al. 2001). In addition, there have been criticisms of the complexity of the study, accompanied by calls for deleting some test components from the protocol (Li 2005) or using screening approaches that incorporate DNT testing into other testing protocols (Cooper et al. 2006; Ladics et al. 2005). Critics also claim that variability of some end points (e.g., motor activity, morphometrics) is too great to be useful (Chemical Manufacturers Association 1987; Nolen 1985; York et al. 2004) and that this *in vivo* test is not necessary to detect DNT (Balls and Combes 2005). These diverse opinions do not invalidate the DNT study but rather highlight the need for ongoing scientifically based evaluation of this test method and the incorporation of appropriate revisions as scientific knowledge advances and as experience with the DNT study warrants.

A number of efforts are currently under way to review data from existing DNT studies, identify ways to refine the DNT test, and, if possible, reduce the number of animals used. It has been proposed that a reduction in animal use can be achieved by applying certain statistical approaches to the behavioral analysis (Chiarotti and Puopolo 2000; Puopolo 2004). Reviews of historical and positive control data have demonstrated the need for more standardized reporting requirements (Crofton et al. 2004, 2008). Further retrospective reviews of control data have identified differences among laboratories in data quality and variability, suggesting methods to decrease variability (Crofton et al. 1991, 2004; Raffaele et al. 2003, 2004; Sette et al. 2004). Conversely, a review of various neuropathology assessments (e.g., brain weight, standard histopathology, and morphometric assessments) identified low variability for these measures (Crofton et al. 2001), concluding that no one postmortem measure is more sensitive, with each providing important data (Raffaele et al. 2005). The outcome of this continuing effort will allow better data interpretation, help refine requirements for future testing, and guide new methods development.

In addition to the goal of refinement of the current approach to DNT testing, there is another and more pressing driver of change in the science arena of DNT. Currently, thousands of chemicals lack even simple, basic toxicologic data (e.g., high-production-volume chemicals, pesticide inert ingredients, antimicrobial pesticides) but have a high potential for human exposure (NRC 1984). Assessing potential neurotoxicologic effects for these chemicals is a major challenge confronting the chemical industry, international and national regulatory agencies, and associated stakeholders (Dix et al. 2007; Kavlock et al. 2008; NRC 2007). New tools and methods are required to move toward a more sustainable risk assessment paradigm for these types of chemicals. Although the current DNT guidelines generate useful data for risk assessment purposes, this *in vivo* test is costly and time-consuming and uses a relatively large number of naive animals when conducted as a stand-alone study (compared with incorporating the DNT testing into other protocols, e.g., as proposed in Cooper et al. 2006 and U.S. EPA 2002b). A pressing goal of future research is to develop a validated true first-tier screening paradigm (e.g., a high-throughput *in vitro* screening battery) that can rapidly screen large numbers of chemicals for their potential to cause DNT (Coecke et al. 2007; Lein et al. 2007; NRC 2007; U.S. EPA 2006). Coupled with development of decision frameworks (e.g., Combes et al. 2003), data from these high-throughput screens may facilitate prioritization of any further testing *in vivo*, for example, as for substances identified as potentially hazardous under the European regulation on Registration, Evaluation, Authorisation, and Restriction of Chemical Substances (REACH) (European Commission 2006). Data generated by the current DNT test method will be vital in the validation of these high-throughput *in vitro* methods, providing information on their utility and limits, as well as guidance on the potential use of data from these alternative methods in a risk assessment context.

## Conclusions

The OECD DNT TG 426 (OECD 2007) represents the best available science for assessing the potential for DNT in human health risk assessment, and data generated by DNT studies are relevant and reliable for this assessment. The test methods used in the DNT have been subjected to an extensive history of international validation, peer review, and evaluation that is contained in the public record. The reproducibility, reliability, and sensitivity of these methods have been demonstrated, using a wide variety of test substances. Multiple, independent, expert scientific peer reviews affirm these conclusions, as described in this document. The OECD DNT TG 426 provides an outline of behavioral domains and morphologic end points, relevant to human neurodevelopment, that should be examined to assess potential DNT of a test compound. The results from DNT studies are used for hazard/risk assessment purposes, and in cases where data from a DNT study are not available, additional uncertainty factors may be employed by regulators to address the need for DNT data from a regulatory standpoint. This document shows that a variety of chemicals have been tested for DNT, constituting a sampled spectrum of the chemical universe that the test is proposed to investigate. Several published reports outlined herein show that the DNT study is robust and can be conducted in multiple laboratories with consistent performance.

## Figures and Tables

**Table 1:** Historical contributions to the DNT guideline.

Date	Event	Summary	References
1960s–1980s	Published research on DNT and behavioral testing	Evidence that developmental exposure to chemicals and drugs can alter behavioral function in young and adult animals	Irwin 1968, Spyker and Smithberg 1972, Barlow and Sullivan 1975, Butcher et al. 1979, Butcher and Vorhees 1979, Vorhees et al. 1979, Butcher and Nelson 1985, Adams 1986
1978–1984	CBTS	Study to examine intra- and interlaboratory reliability and sensitivity of behavioral test methods	Buelke-Sam et al. 1985, Kimmel and Buelke-Sam 1985, Kimmel et al. 1985
1984	Cincinnati Test Protocol	Within-laboratory comparison of CBTS test protocol with the Cincinnati Test Protocol	Vorhees 1985a, Vorhees 1985b, Vorhees 1985c
1982–1985	Collaborative studies of the Japanese Teratology Society	Interlaboratory methods evaluations and assessment of six reference chemicals	Tanimura 1986, Tanimura 1992
1985–1988	European Interlaboratory Collaborative Study	Interlaboratory study to assess sensitivity of behavioral test procedures to detect neurotoxicity of methylmercury	Elsner 1986, Elsner et al. 1986, Suter and Schon 1986
1989	Williamsburg Workshop	Workshop to evaluate the qualitative and quantitative comparability of animal and human data for DNT	Francis et al. 1990, Kimmel et al. 1990
1993–1997	Collaborative studies of the Japanese Teratology Society	Three interlaboratory studies using behavioral teratogens comparability a core battery of tests	Tachibana et al. 1996, Tachibana et al. 1998, Fukunishi et al. 1998, Nishmura et al. 2001
1995	IPCS	Interlaboratory study using neurotoxic chemicals to evaluate test validity, reliability, and measurement variability	Catalano et al. 1997, MacPhail et al. 1997, Tilson et al. 1997
2000	ILSI workshop on DNT testing	Workshop to review U.S. EPA DNT behavioral test methods, pharmacokinetics, and neuropathology	Cory-Slechta et al. 2001, Dorman et al. 2001, Garman et al. 2001, Mileson and Ferenc 2001
2003	Japanese Interlaboratory Study	Interlaboratory study using neurotoxic chemicals to determine sensitivity of behavioral measures	Okazaki et al. 2003
2003	Behavioral Test Methods Workshop	Expert workshop to address design, conduct, and analysis of behavioral tests for neurotoxicity evaluation	Slikker et al. 2005
2004–2008	ILSI RSI Working Group	Working group focused on variability, statistical analyses, positive controls, identification and analyses, interpretation of treatment-related effects, and application of DNT testing to public health protection	Fenner-Crisp et al. 2005, Crofton et al. 2008, [Bibr b47-ehp-117-17][Bibr b102-ehp-117-17],Tyl et al. 2008

**Table 2:** History of the DNT guideline.

Date	Event	Reference
1986	U.S. EPA OPPTS published first draft DNT protocol for peer review and public comment	U.S. EPA 1986
1991	U.S. EPA OPPTS published final DNT guideline (§83–6)	U.S. EPA 1991
1995	OECD Working Group on Reproduction and Developmental Toxicity (Copenhagen) recommended development of OECD Developmental Neurotoxicity Test Guideline	OECD 1995
1996	OECD expert consultation meeting (Copenhagen) provided recommendations for design of Draft OECD 426	OECD 1996a
1998	U.S. EPA OPPTS issued minor revisions and harmonization of DNT guideline (OPPTS 870.6300)	U.S. EPA 1998
1998	Draft TG 426 submitted to National Coordinators for expert review and comment	
2000	OECD expert consultation meeting (Washington) held to review technical issues	OECD 2003
2003	Draft TG 426 submitted to National Coordinators for expert review and comment	
2005	OECD expert consultation meeting (Tokyo) convened to respond to remaining comments on Draft TG 426	OECD 2005b
2007	OECD TG 426 approved by WNT; guideline finalized	OECD 2007

WNT, the working group of the National Coordinators of the Test Guidelines Programme.

**Table 3:** Number of chemicals studied using the U.S. EPA DNT guideline or draft OECD DNT guideline.

Chemical class	No. of studies
Industrial chemicals	8
Miscellaneous agents^a^	4
Pharmaceuticals	3
Pesticides	73
Positive control chemicals	15
Solvents	7

a Food additives, cigarette smoke, dietary restriction, and maternal separation.

**Table 4:** Examples of chemicals and other stressors tested using the U.S. EPA DNT guideline or OECD TG 426.^a^

Abamectin	Dichlorvos (2)	Methyl parathion
Acephate	Dicrotophos	Methylazoxymethanol (2)
Acetamiprid	Dietary restriction	Methylmercury
Acibenzolar-*s*-methyl	Dimethoate	*n*-Methylneodecanamide
Acrylamide	Disulfoton	Molinate
AE-0172747	Emamectin	Naled
Aldicarb	Epidermal growth factor	Nelfinavir
Alitame	*s*-Ethyldipropylthiocarbamate	Nitrous oxide
Amicarbazone	Ethoprophos	Octamethylcyclotetrasiloxane
Atorvastatin	Ethylbenzene	Perchlorate
Azinphos methyl	Etofenprox	Phorate (2)
BAS 510F	Fenamidone	Prochloraz
BAS 670H	Fenamiphos	Profenofos
Bifenthrin	Fentin hydroxide	Propylthiouracil (2)
Carbaryl	Fipronil	Pymetrozine
Carbofuran	Flubendiamide	Pyrasulfotole
Chlorfenapyr	Flufenacet	Spirodiclofen
Chlorite, sodium	Glufosinate ammonium	Prothioconazole
Chlorpyrifos	Glyphosate trimesium	Styrene
CI-943	GN1180 (MN rgp120/HIV-1)	Tetrabromobisphenol A
Cigarette smoke	Hydrogen sulfide	Tebuconazole
Clodinafop propargyl	Imidacloprid	Terbufos
Clothianidin	Iminodiproprionitrile	Tetrachlorvinphos
Coumaphos	Indoxacarb	Thiamethoxam
Cyclohexanemethanol	Isopropanol	Thiocloprid
λ-Cyhalothrin	Isoxaflutole	Thiram
β-Cyfluthrin	Lead nitrate	Triallate
Cymoxanil	Lindane	Tribufos
ϑ-Cypermethrin	Malathion	Trichlorfon
Decamethylcyclopentasiloxane	Maternal separation	1,1,1-Trichloroethane
*p,p*-DDT	Methamidaphos	Trichloroethylene
DEET (*N*,*N*-diethyl-*meta*-toluamide)	*p*-Methane-3,8-diol	Triethylene glycol monomethyl ether
Deltamethrin	Methimazole (6)	Trimethyltin
Diazepam	Methyl bromide	Ziram
Diazinon		

a Numbers indicate the number of studies conducted for that chemical
